# Ethnic Comparisons of Spike-Specific CD4+ T Cells, Serological Responses, and Neutralizing Antibody Titers Against SARS-CoV-2 Variants

**DOI:** 10.3390/vaccines13060607

**Published:** 2025-06-04

**Authors:** Fani Pantouli, Vanessa Silva-Moraes, Ted M. Ross

**Affiliations:** 1 Florida Research and Innovation Center, Cleveland Clinic, 9801 SW Discovery Way, Port Saint Lucie, FL 34987, USA; pantouf@ccf.org (F.P.); moraesv@ccf.org (V.S.-M.); 2 Department of Infection Biology, Lerner Research Institute, Cleveland Clinic, Cleveland, OH 44195, USA; 3 Center for Vaccines and Immunology, University of Georgia, Athens, GA 30602, USA; 4 Department of Infectious Diseases, University of Georgia, Athens, GA 30602, USA

**Keywords:** COVID-19, SARS-CoV-2 variants, spike, ethnicity, AIM+ CD4+ and CD8+ T cells, activation markers, memory cells, neutralizing antibody titers, RBD-IgG antibody titers, cytokines

## Abstract

Background/Objectives: To evaluate how immune responses compare among ethnic groups approximately 2 years after receiving a third dose of COVID-19 vaccine (BNT162b2, mRNA-1273, ChAdOx1or BBIBP-CorV), we tested T cell responses and Spike-specific RBD-antibody titer, and neutralized antibody titer levels utilizing Spectral Flow cytometry, ELISA, and SARS-CoV-2 pseudotyped-based neutralization assays, respectively. Methods: Forty-four individuals from January–December 2023 were identified within the cohort and were classified into different ethnic backgrounds; Black (N = 13), Asian (N = 14), Caucasian (N = 17). We recognize that the “Asian” group includes diverse subpopulations with distinct genetic and environmental backgrounds, which could not be further stratified due to sample-size limitations. Spike-specific AIM+, CD4+, and CD8+ T cell responses were assessed and evaluated against SARS-CoV-2 variants, including the ancestral Wuhan, Delta, and multiple Omicron subvariants (B1.1529, BA2.86, BA.4/5, and XBB.1). Alongside we tested the RBD-IgG and neutralizing antibody titers against the ancestral Wuhan. Spearman’s correlation analysis was utilized to determine corelative relationships among the AIM+ and CD4+ T cell responses, as well as the RBD-IgG and neutralizing antibody titers. Results: Our results show robust and comparable RBD-IgG and neutralizing antibody titers across all groups, with a significant positive correlation between these two measurements. Significant differences were observed in T-cell activation, with Asian participants exhibiting lower frequencies of Spike-specific CD4+ T cells against SARS-CoV-2 Omicron subvariants and higher frequencies of cytokine-producing CD4+ T cells (TNF-α, IFN-γ, and IL-2) as compared to the Caucasian group. Breakthrough infection status was not fully controlled and may influence these findings. Conclusion: Despite a small sample size and potential confounding by natural infections within our long-time-span sampling, our data suggest persistent cellular and humoral immunity 2 years after vaccination across ethnicities, with notable differences in T cell activation and cytokine profile. These preliminary observations highlight the need for larger, more detailed studies that consider intra-ethnic diversity and hybrid immunity to better understand ethnic differences in COVID-19 vaccine responses.

## 1. Introduction

COVID-19 vaccines effectively induce SARS-CoV-2-specific neutralizing antibodies [1] and CD4+ and CD8+ T cell responses [2,3]. These immune responses may vary across different ethnic populations due to genetic diversity and dietary factors. Ethnic dietary patterns affect immune function by regulating nutrient availability, inflammation, and the gut microbiome [4]. Diets rich in micronutrients, for example, vitamins D, A, C, zinc, and polyunsaturated fats, may enhance immunity, whereas western-style diets may impair it [4,5]. Such differences may contribute to observed variations in vaccine-induced responses between ethnic groups [6]. African Americans were the most likely to be seropositive to COVID-19, and Asian Americans face a higher risk of intensive care unit admission due to COVID-19 compared to Caucasian patients. These might be attributed to higher rates of comorbidities and socioeconomic factors within these groups and influence the varying levels of vaccine protection against severe COVID-19 [7]. Currently, the updated monovalent omicron KP.2-derived vaccine is available and remains effective against SARS-CoV-2 infections, despite the emergence of new SARS-CoV-2 variants [8]. Meanwhile, reinfections are common, resulting in milder disease and reduced hospitalization rates [9]. Most individuals worldwide are no longer immunologically naive to SARS-CoV-2, having acquired immunity through infection, vaccination, or both.

Memory T cells play a crucial role in this immunity. The lack of long-term sterilizing immunity and the unpredictable nature of SARS-CoV-2 evolution raise concerns about potential resurgences of infections and hospital admissions [10]. Circulating antibody levels against SARS-CoV-2 persist for at least 6 months and start declining faster than T cell responses, offering limited cross-protection against variants [3,11,12,13]. Meanwhile, CD4+ and CD8+ T cells are maintained approximately 6–7 months after three vaccination doses [2] and significantly contribute to ongoing protection against severe outcomes and hospitalization [3,13]. Understanding the long-term development of T cell responses to SARS-CoV-2, including memory and activation, and their correlation with receptor-binding domain (RBD) antibody titers or neutralizing antibody titers in various ethnic groups is necessary.

In this study, differences in circulating activated CD4+ and CD8+ memory T cells among Black, Asian, and Caucasian subjects approximately 2 years after receiving a third dose of a COVID-19 vaccine (BNT162b2, mRNA-1273, ChAdOx1, or BBIBP-CorV) were investigated. We acknowledge that the “Asian” cohort was treated as a single group due to sample-size limitations, potentially masking intra-ethnic variability related to genetics, diet, and environment. The immune responses against various SARS-CoV-2 variants, including the ancestral Wuhan strain, Delta, and several Omicron subvariants (B1.1.529, BA2.86, BA4/5, XBB.1), were assessed. Additionally, RBD-IgG antibody titers and neutralizing antibody titers were measured, both of which are indicators of vaccine protection. Finally, correlative relationships were assessed between RBD antibody levels, neutralizing antibody levels, and activated T cells within these groups. This study demonstrated that AIM+ and CD4+ T cell responses are decreased, while T helper type 1 (Th1) cytokine levels are increased against SARS-CoV-2 Omicron subvariants in Asian descent individuals compared to Caucasians. Our findings provide preliminary insights into the durability and variability of vaccine-induced immunity across ethnic groups but highlight the need for larger studies that stratify ethnic subpopulations and control for natural infection status.

## 2. Materials and Methods

### 2.1. Study Participants

**Figure 1:**
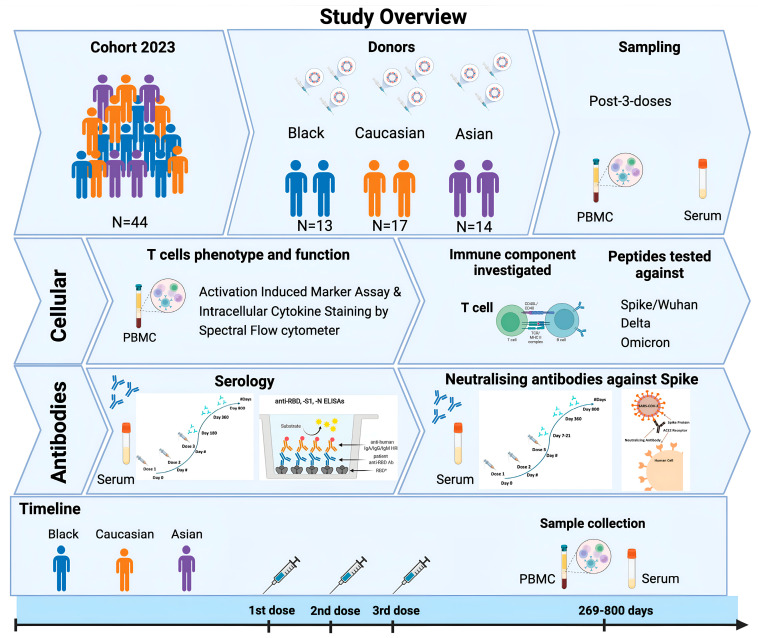
Schematic of study summary. Forty-four participants from the SPARTA 2023 cohort had received three consecutive doses against SAR-CoV-2 and were selected for the completion of the study, including individuals from Black (N = 13), Caucasian (N = 17), and Asian descent (N = 15). Samples (PBMCs, sera) from those individuals were collected after 269–800 days from their third vaccination and were further processed for T cell immunophenotyping under Spectral Flow cytometry and for neutralizing antibody assays against Spike/Wuhan. Serum samples from earlier time points (7–21 days post-3rd dose) were also tested for their neutralizing antibody titers against Spike/Wuhan using unpaired individuals for comparison. Serology was performed in the same individuals by detecting the RBD-antibody titer levels longitudinally from a period of 6 months until 2 years post-vaccination, subdivided into post-second and post-third vaccination booster doses.

The SPARTA (SARS SeroPrevalence and Respiratory Tract Assessment) program, funded by the U.S. National Institutes of Health, investigates the immune responses triggered by SARS-CoV-2 infection and/or COVID-19 vaccination. Participants between the ages of 22 and 65 years were recruited starting in October 2020 with written informed consent obtained in Athens (GA, USA) and Port Saint Lucie (FL, USA). The study procedures, informed consent, and data-collection documents received approval from the WIRB Copernicus Group Institutional Review Board (approval no. 20202906) [14,15]. Forty-four individuals were identified within the cohort, including one-to-two individuals per group with pre-existing immunity to SARS-CoV-2 (previous SARS-CoV-2 infection before vaccination). The other participants were immunologically naïve to the virus (no SARS-CoV-2 infection before vaccination) based on Ag test (PCR), self-reporting, and/or anti-receptor-binding domain (RBD) antibody levels. Some participants during the study had breakthrough infections (infections in 2021 after they had received their third vaccination), and they were identified based on the same criteria. Samples were collected between 269 and 800 days after the third Wuhan mRNA vaccination dose. Participants from different racial/ethnic backgrounds were classified as either Black (N = 13), Asian (N = 14), or Caucasian (N = 17). Most Caucasian participants were under the Caucasian/Non-Hispanic/Non-Latino subdivisions, with three individuals categorized as Caucasian/Hispanic or Latino. However, this group is referred to as Caucasian. Participants received three vaccinations of Pfizer BNT162b2 monovalent, the bivalent or Moderna mRNA vaccines, the chadox1 ncov-19 (AstraZeneca, Cambridge, UK), or BBIBP-CorV (Sinopharm, Beijing, China). Any reported history of chronic health conditions or prescribed medications during the study observation is outlined in Table 1. Demographics, including race, gender, vaccine-type distribution, and clinical characteristics, are summarized in Table 1, and for an analytical outline of the characteristics of each participant, see Table A1. For a study overview, see Figure 1.

### 2.2. Whole Blood Processing and Enzyme-Linked Immunosorbent Assay (ELISA)

Whole blood processing was performed as previously described [16], and ELISA was conducted according to the established protocols [14,16,17].

### 2.3. Flow Cytometry

Peptides

Peptides were obtained in collaboration with La Jolla Institute for Immunology (CA, USA). The Sette lab has previously developed the megapool approach to allow simultaneous testing of large number of epitopes, as previously reported [18,19,20]. According to this approach, large numbers of different epitopes are solubilized, pooled, and re-lyophilized to avoid cell toxicity problems associated with high concentrations of DMSO typically encountered when single pre-solubilized epitopes are pooled. Previous work has described the preparation of the peptides from Wuhan, Delta, B1.1.529, BA2.86, BA.4/5, and XBB.1 sequences that were made at 1 mg/mL [21].

Activation-induced marker and intracellular cytokine-staining assays

**Figure A1:**
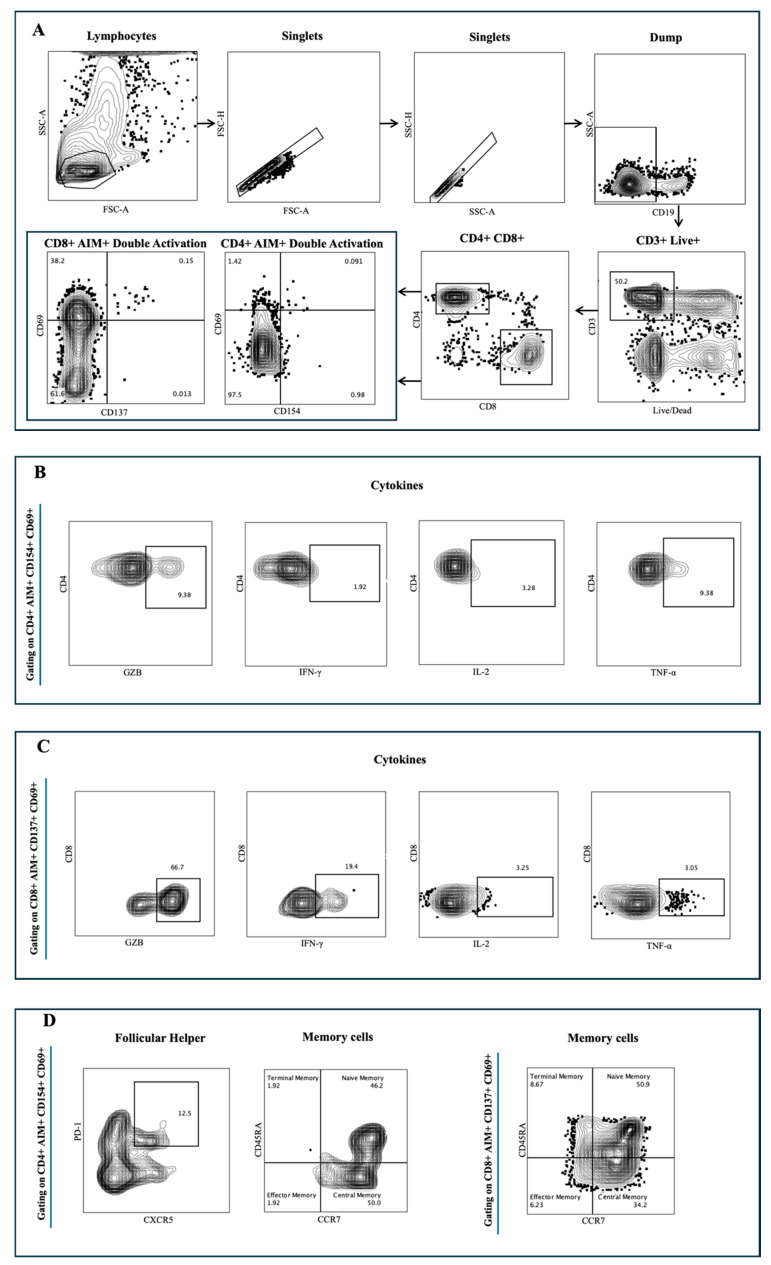
Gating strategy diagram. (**A**) Gating from lymphocytes to Singlets to CD3+/Live+ and further gating on CD4+ and CD8+ T cell subsets. Further gating on each of these populations for double activation defined as CD154+ CD69+ CD4+ T cells and CD137+ CD69+ CD8+ T cells. (**B**) Cytokines such as GZB, IFNγ, IL-2, TNF-α are gated on the (**B**) CD154+ CD69+ CD4+ T cells and, (**C**) CD137+ CD69+ CD8+ T cells. (**D**) Additional gating was performed on T follicular Helper cells defined as CXCR5+ PD1+ inside CD154+ CD69+ CD4+ T cells, memory cells defined as CD45RA− CCR7+ for central memory; CD45RA+ CCR7+ for naïve memory; CD45RA−CCR7− for effector memory and CD45RA+ CCR7− for terminal memory inside CD154+ CD69+ CD4+ T cells and CD137+ CD69+ CD8+ T cells.

SARS-CoV-2 spike-specific T cell responses were measured for all donors at all available time points utilizing two previously described flow cytometry methods: activation-induced marker (AIM) assays and intracellular cytokine staining (ICS) with modifications [16,21]. Peripheral Blood Mononuclear Cells (PBMCs) were thawed by warming frozen cryovials in a 37 °C water bath and resuspending cells in 10 mL of culture media (RPMI-1640 media containing 2 mM L-glutamine, sodium bicarbonate, and supplemented with 10% FBS, 1% penicillin/streptomycin, 2-ME, sodium pyruvate, and nonessential amino acids) in the presence of Benzonase (50 U/mL, Millipore #70664-3). Cells were centrifuged at 500× *g* for 10 min, and cells were resuspended in media containing 100 ug/mL DNAse. Cells were counted (LUNA-II^TM^ cell counter, Logos Biosystems, Anyang, Republic of Korea) and rested in the same media for 1 h at 37 °C, 5% CO_2_. A subsequent wash was performed, and cells were resuspended at a 2 × 10^6^ cells/mL density into media containing 25 μg/mL DNAse and were left to rest overnight (8–15 h) at 37 °C, 5% CO_2_. The next day, cells were washed, resuspended in culture without DNAse, and plated at a density of 1 × 10^6^ cells/mL/well/50 μL in U-bottom 96-well plates. Next, cells were incubated in 50 μL/well of anti-CD40 monoclonal Antibody (Miltenyi Biotec, San Diego, CA, USA) at a final concentration of 0.5 mg/mL at 37 °C, 5% CO_2_ for 15 min. Following blocking with anti-CD40, 50 μL of Spike-specific peptide pool (Wuhan, China) or Delta or B1.1.529 or BA2.86 or BA.4/5 or XBB.1 (1 mg/mL/peptide final concentration) was added in a final volume of 150 μL. Stimulation with an equimolar amount of DMSO was performed as a negative control, and phytohemagglutinin (PHA) (1 mg/mL, Millipore, Burlington, MA, USA) (Cat#EMD431784) was included as positive control. Plates were left to incubate at 37 °C, 5% CO_2_ for 20–24 h. After 22–24 h, the protein transport inhibitor Golgi-stop and Golgi-plug (eBioscience, San Diego, CA, USA) were added at 50 μL/well for a final volume of 200 μL/well and were left to incubate for an additional 4 h at 37 °C, 5% CO_2_. Upon the incubation completion (26–28 h), plates were centrifuged at 705× *g* for 10 min at room temperature, and cells were resuspended in 200 μL PBS + EDTA 2 mM for 5 min at room temperature. Cells were washed with PBS and incubated for 20 min at room temperature with live/dead (NIR, no. L10119, Thermo Scientific, Waltham, MA, USA) and Fc receptor blocking solution (human BD Fc Block, BD Biosciences, Franklin Lakes, NJ, USA Cat#564220) in PBS (50 μL/well). Cells were spun again, resuspended, and 50 μL/well of surface antibodies diluted in FACS buffer (PBS supplemented with 0.5% BSA and 2 mM EDTA) were added and incubated for 30 min in the dark at 4 °C. Cells were washed twice with FACS buffer and fixed for 20 min at 4 °C in the dark (CytoFix/CytoPerm, Cat no. 52-2090K2, BD Biosciences, Franklin Lakes, NJ, USA). For the ICS process, cells were washed twice with BD Perm/Wash buffer (Cat No. 51-2091K2) and centrifuged at 1100× *g* for 6 min at room temperature. Cells were then incubated in Perm/Wash buffer for 5 min at room temperature and washed again. The intracellular antibodies solution (50 μL/well) was added, and cells were incubated for 30 min at room temperature. Next, cells were washed once with perm/wash buffer, followed by a wash in FACS, and finally resuspended in FACS for data acquisition. Antigen-specific CD4+ and CD8+ T cells were measured as a percentage of AIM+ cells (CD154+, CD69+ and CD137+, CD69+, respectively). Antigen-specific memory subsets were defined as a percentage of AIM+, CD4+, and CD8+ T cells expressing CD45RA and CCR7 markers: CD45RA−-CCR7+ (central memory), CD45RA+ and CCR7+ (naïve), CD45RA+ and CCR7− (terminal effector memory), and CD45RA− and CCR7− (effector memory). Antigen-specific circulating T follicular helper cells (cTfh) were defined as a percentage of AIM+ T cells expressing (CXCR5+ PD1+). For the ICS staining, proinflammatory cytokines such as Granzyme B (GZB), Interferon-gamma (IFNγ), Tumor necrosis factor alpha (TNFα), and Interleukin (IL-2) were measured as a percentage of antigen-specific AIM+, CD4+, and CD8+ T cells (CD154+, CD69+ and CD137+, CD69+, respectively). The gating strategy is available in Figure A1. All samples were acquired on the Cytek Aurora Spectral Flow cytometer system with four lasers. The list of antibodies used in this panel can be found in Table A2. CD4+ and CD8+ T cell counts were determined by subtracting the count from each paired unstimulated (DMSO-treated) control sample (stimulated-paired unstimulated). The Ag-specific AIM+ subsets were measured as background (DMSO-treated) subtracted data, with a minimal DMSO level set to 0.005, and a response greater than that was considered positive.

### 2.4. SARS-CoV-2 Pseudolentivirus and Neutralization Assay

To generate lentiviral particles with SARS-CoV-2 Spike proteins for use in in vitro neutralization assays, HEK-293 cells were cultured at 10^6^ cells/mL density in a T175 flask a day before being transfected according to Lipofectamine^TM^ 3000 (Thermo-Fisher Scientific, Carlsbad, CA, USA) protocol using the following plasmid constructs; luciferase-IRES-ZsGreen lentiviral backbone (NR-52516), Gag-pol HMD-Hgpm2 (NR-52517), Rev1b pRC-CVM-Rev1b (NR-52519), Tat1b HDM-tat1b (NR-52518), and the viral entry protein of the full-length pHDM SARS-CoV-2 Spike Wuhan (NR-52514) to produce the SARS-CoV-2 Spike lentivirus. All plasmids were obtained from BEI Resources. Following 72 h incubation in OptiMEM transfection solution at 37 °C, supernatants were harvested and concentrated by centrifugation using an Amicon 15 mL and stored in aliquots at −80 °C until further usage.

The neutralization activity of serum samples against the SARS-CoV-2 virus/ancestral Wuhan strain was adapted as previously described [15,22]. HEK-293 cells overexpressing the Angiotensin-converting enzyme 2 (ACE2) cell surface receptor were cultured in Dulbecco’s Modified Eagle Medium (DMEM) supplemented with 10% FBS and were seeded at 200,000 cells/mL/well density in poly-L-lysine (Sigma, Cream Ridge, NJ, USA) pre-coated 96-well culture plates. Following 12–24 h after plating, 50 μL of heat-inactivated sera samples (at 56 °C for 30 min) at threefold serial dilutions were pre-incubated with an equal volume of the pseudotyped SARS-CoV-2 Spike lentiviruses containing approximately 200,000 relative light units (RLU)/50 μL/well at 37 °C, 5% CO_2_ for 1 h. After the incubation completion, sera/virus mixtures were added to the 96-well plate containing the HEK-293 ACE2 pre-seeded cell monolayer, and plates were incubated for 1 h at 37 °C, 5% CO_2_. Next, the serum–virus mixture was removed, and 100 μL of DMEM supplemented with 2% FBS were added to the wells and left to incubate for 48 h. Two days later, cells were incubated in equal volume of Bright-Glo^TM^ Luciferase Assay system (Cat no. E2650, Promega, Madison, WI, USA) at a 1:1 ratio for 5 min in the dark at room temperature. Cells were then transferred to an opaque 96-well plate for reading at the luminometer (Promega). All samples were assessed in duplicates starting at a 1:10 dilution and then three-fold serially diluted. Internal controls used included cell-only wells and pseudovirus-only wells. The average cell-only RLUs were defined as 100% of virus neutralization, while the average mean virus-only RLU was defined as 0% neutralization. Antibody titer was then estimated by interpolating the point at which infectivity had been reduced to 50% of the value for the no-serum control samples. The samples tested average RLU values, which were normalized to both virus control and cells only. The resulting IC50 values represent PsVN50 for the tested sera. Samples that failed to neutralize SARS-CoV-2 at a dilution of less than 1:20 were accepted as 20, which defined the limit of detection, and any sample that did not neutralize SARS-CoV-2 at a 20-fold dilution was assigned a value of 20 for representation and data-analysis purposes. Data analysis was performed as previously described [23].

### 2.5. Statistical Analysis

GraphPad Prism Version 10 (GraphPad Software Inc., San Diego, CA, USA) was used to perform all statistical analyses. Statistical comparisons between participant groups (Black, Asians, Caucasians) were made using unpaired Mann–Whitney non-parametric T-tests for the cellular responses. Correlative analysis was performed using Spearman’s correlation coefficient. For the serology results, two-way ANOVA multiple comparisons with Tukey post-hoc analysis were utilized for factors Ethnicity, Days, and Ethnicity × Days Interaction. For the neutralization assay, RLU values were utilized to determine PsVN50 titers alongside their corresponding serum dilutions through nonlinear fitting of inhibitor vs. normalized response of inhibitor concentration 50 (IC50). Kruskal–Wallis with Dunn’s multiple comparisons test was utilized to compare the three timepoints from the neutralization assay data. The statistical details of the experiments are provided in the respective figure legends. Data are plotted as the median and 95% confidence limits (95 CI). Details denoting significance will be found in the respective legends. The value of *p* < 0.05 was considered statistically significant.

### 2.6. Ethical Approval

The studies involving humans were approved by WIRB and University of Georgia IRB #20202906. The studies were conducted in accordance with the local legislation and institutional requirements. The participants provided their written informed consent to participate in this study.

## 3. Results

Forty-four participants from the SPARTA cohort that received three consecutive COVID-19 vaccinations against SARS-CoV-2 were selected and separated by three ethnic categories: Black (N = 13), Caucasian (N = 17), and Asian (N = 14) (Figure 1).

PBMC and serum samples were collected 269–800 days following the third vaccination and were further processed for T cell immunophenotyping against different variants as described above, and their neutralizing activity against Spike/Wuhan. Neutralizing antibody titers were also assessed at the time of enrollment (baseline) and at 7 to 21 days following the third vaccination (Figure 1) for comparison with earlier time points. Most participants had no previous infection with the SARS-CoV-2 (Wuhan) between July 2020 and January 2021. One-to-two participants per group had previously been infected with SARS-CoV-2 (Table 1). All 44 of these participants received either the COVID-19 mRNA vaccine (Pfizer, New York, NY, USA, Pfizer Bivalent, Moderna, Norwood, MA, USA), the inactivated virus vaccine with an adjuvant (Sinopharm), or vector vaccine (AstraZeneca). There were approximately twice as many female participants as male participants in each ethnic group (Table 1).

Several participants had breakthrough infections following their third vaccination. Demographic information is in Table 1, and for an extensive description per participant, see Table A1.

### 3.1. SARS-CoV-2 Spike-Specific AIM+ CD4+ and CD8+ T Cell Responses

**Figure 2:**
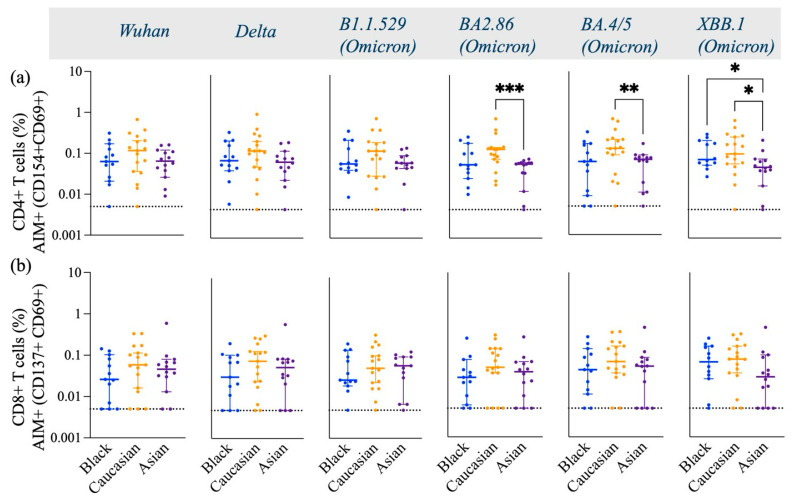
Frequency of Spike-specific AIM+ (CD154+ and CD69+) CD4+ T cells and AIM+ (CD137+ and CD69+) CD8+ T cells across Wuhan, Delta, and Omicron subvariants among Black, Caucasian, and Asian participants. (**a**) Mann–Whitney unpaired *t*-test showed no difference in Spike-specific response among Wuhan, Delta, and Omicron/B1.1.529-specific CD154+ CD69+ CD4+ T cells. Yet significantly lower frequencies were detected in Asian participants as compared to the Caucasians against Omicron/BA2.86 (*p* < 0.001), Omicron/BA4.5 (*p* < 0.01), and Omicron/XBB.1 (*p* < 0.05). Additionally, the Asian group had significantly lower frequencies as compared to Black participants (*p* < 0.05). (**b**) Mann–Whitney unpaired *t*-test showed no difference among the groups (*p* > 0.05) when evaluated against Wuhan, Delta/B1.617.2, Omicron/B1.1.529, Omicron/BA2.86, Omicron/BA4.5, and Omicron/XBB.1-specific CD137+ CD69+ CD8+ T cell responses. Data are represented as Median ± 95 CI for the Black (N = 12–13), Caucasian (N = 17), and Asian (N = 14) participants. Significance is noted as *p* < 0.05 *, *p* < 0.01 **, *p* < 0.1 ***.

The AIM and ICS flow cytometry assays were utilized to investigate the long-term effect of SARS-CoV-2 spike-specific CD4+ T cell responses induced by vaccination [24]. PBMCs were stimulated with peptide pools spanning the Spike sequence from the Wuhan, Delta, and Omicron subvariants isolated from 2020 to 2023. Overall, most participants had increased Spike-specific CD4+ T cell response across all strains tested 269–800 days following their last vaccination (Figure 2).

**Figure 3:**
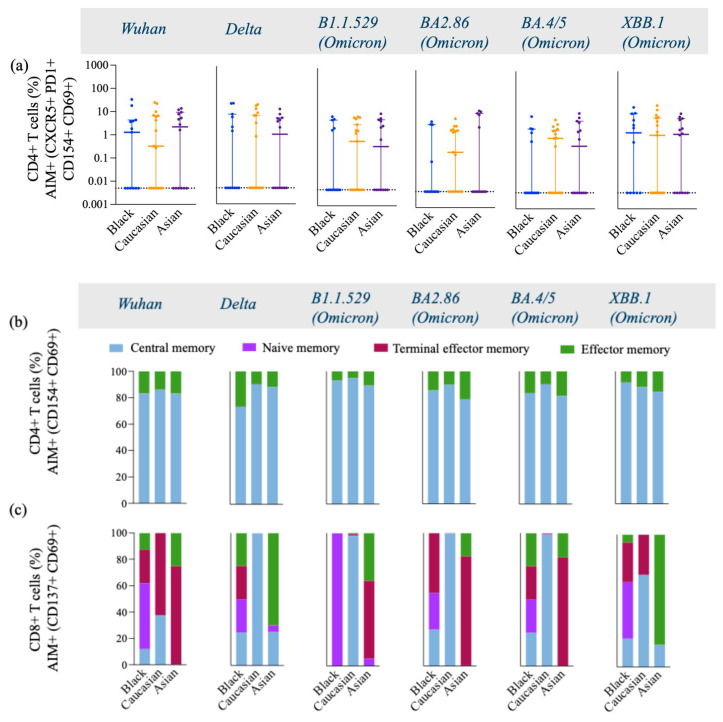
Frequency of T follicular Helper cells and AIM+, CD4+, and CD8+ Memory T cell subtypes gated on AIM+ and CD4+ double-activated T cells across variants from Wuhan, Delta, and Omicron subvariants among Black, Caucasian, and Asian participants. (**a**) Mann–Whitney unpaired *t*-test showed no difference among the groups (*p* > 0.05) for Wuhan, Delta/B1.617.2, Omicron/B1.1.529, Omicron/BA2.86, Omicron/BA4.5, and Omicron/XBB.1–specific CXCR5+, PD1+, CD154+, CD69+, and CD4+ T cell responses. Data are represented as Median ± 95 CI for the Black (N = 12–13), Caucasian (N = 17), and Asian (N = 14) participants. (**b**,**c**) AIM+ and CD4+ memory cells were gated on CD154+, CD69+, and CD4+ T cells and showed greater predominance for the expression of central and effector memory cell subtypes across all variants within all three groups, with a similar distribution pattern among the groups. AIM+ and CD8+ memory cells were gated on CD137+, CD69+, and CD8+ T cells and showed great diversity in the expression of memory cell subtypes, including central, effector, naïve, and terminal memory cells. Black participants show a diverse memory subtypes repertoire, including all four subtypes across all variants tested, whereas Caucasian participants had a profile of central and terminal effector memory predominantly expressed across all variants. The Asian participants show a higher expression of effector and terminal effector memory cell subtypes across all variants. AIM+, CD4+, or CD8+ memory T cells were defined by the combination of CD45RA and CCR7 markers, where central memory is CD45RA− and CCR7+, naïve memory is CD45RA+ and CCR7+, terminal effector memory is CD45RA+ and CCR7−, and effector memory is CD45RA− and CCR7−, while each population is expressed as % of the total cells.

Spike-specific CD4+ T cells against Wuhan, Delta, and early Omicron variants (B1.1.529) displayed similar frequencies in Caucasian, Black, and Asian participants (*p* > 0.05). However, the frequencies of CD4+ T cells specific to the later Omicron variants were significantly lower in Asian participants compared to Caucasian (BA2.86, *p* < 0.001; BA4.5, *p* < 0.01; XBB.1, *p* < 0.05) (Figure 2a). Additionally, Black participants also had a significantly higher frequency of Spike-specific CD4+ T cells against the XBB.1 strain (*p* < 0.05) compared to Asians (Figure 2a). The frequencies of cTfh were less robust yet comparable among the groups across all variants tested (*p* > 0.05) (Figure 3a). AIM+ and CD4+ effector and central memory T cells displayed a strong response across all ethnic groups, in contrast to their naïve and terminal effector memory subpopulations (Figure 3b).

**Figure 4:**
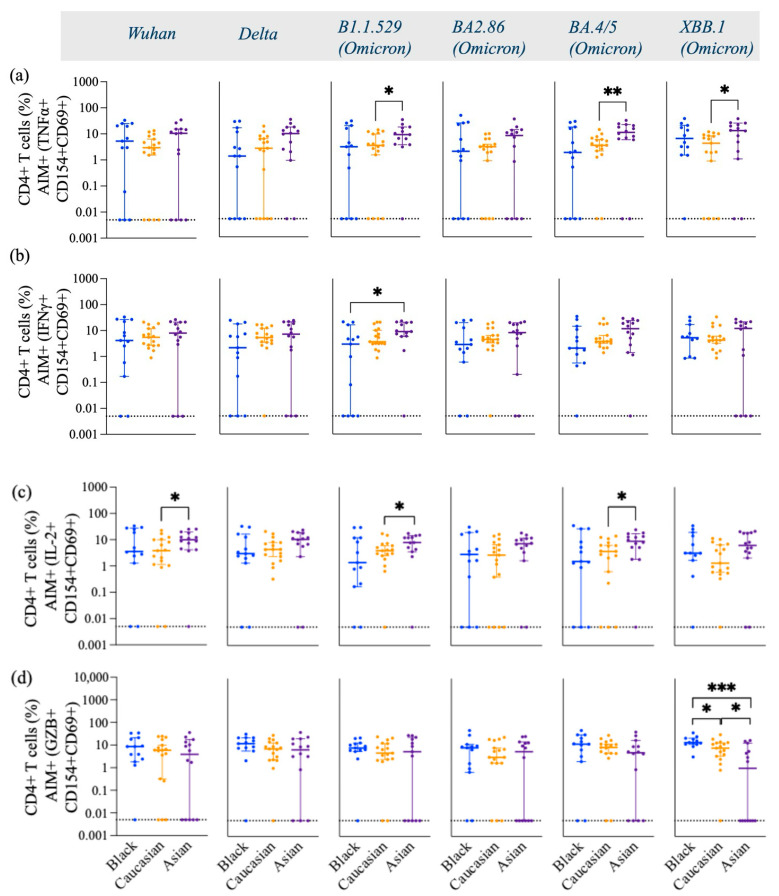
Frequency of TNFα+, IFNγ+, IL-2, and GZB+ gated on AIM+ (CD154+ and CD69+) CD4+ T cells across variants from Wuhan, Delta, and Omicron subvariants—specific among Black, Caucasian, and Asian participants. (**a**) Mann–Whitney unpaired *t*-test showed no difference among the groups for Wuhan and Delta/B1.617.2-specific TNFα+, CD154+, CD69+, and CD4+ T cells. The frequency of Omicron/B1.1.529-specific TNFα+, CD154+, CD69+, and CD4+ T cells was significantly increased in the Asian group as compared to the Caucasian participants (*p* < 0.05). Omicron/BA2.86-specific TNFα+ CD154+ CD69+ CD4+ T cell responses showed no difference among the groups (*p* > 0.05). Omicron subvariants BA4.5 (*p* < 0.01) and XBB.1 (*p* < 0.05)-specific TNFα+, CD154+, CD69+, and CD4+ T cell expression showed significant increase for the Asian participants as compared to the Caucasians. (**b**) Mann–Whitney unpaired *t*-test showed no significant difference among the groups (*p* > 0.05) for Wuhan-specific and Delta/B1.617.2-specific IFNγ+, CD154+, CD69+, and CD4+ T cell responses. Significant increase was detected in the Asian group as compared to the Black participants’ group (*p* < 0.05) for Omicron/B1.1.529-specific IFNγ+, CD154+, CD69+, and CD4+ T cell responses. No significant differences were detected amongst the groups when evaluated against the Omicron subvariants BA2.86, BA4.5, and XBB.1-specific IFNγ+, CD154+, CD69+, and CD4+ T cell responses. (**c**) Mann–Whitney unpaired *t*-test showed significant difference between the Caucasian and the Asian groups for Wuhan-specific (*p* < 0.05), but no difference was detected among the groups (*p* > 0.05) when assessed for Delta/B1.617.2-specific IL-2+, CD154+, CD69+, and CD4+ T cell responses. Significant increase was detected in the Asian group (*p* < 0.05) for Omicron/B1.1.529-specific IL-2+, CD154+, CD69+, and CD4+ T cell responses when compared to the Caucasian participants. No difference was detected among the groups (*p* > 0.05) for Omicron/BA2.86-specific IL-2+, CD154+, CD69+, and CD4+ T cell responses. Significant increase was noted in the Asian group (*p* < 0.05) for Omicron/BA4.5-specific IL-2+, CD154+, CD69+, and CD4+ T cell responses when compared to the Caucasian participants. No significant differences were detected among the groups (*p* > 0.05) when testing for Omicron/XBB.1-specific IL-2+, CD154+, CD69+, and CD4+ T cell expression. (**d**) Mann–Whitney unpaired *t*-test showed no difference among the groups (*p* > 0.05) for Wuhan, Delta/B1.617.2, and Omicron subvariants, as well as B1.1.529, BA2.86, and BA4.5-specific GZB+, CD154+, CD69+, and CD4+ T cells. Mann–Whitney unpaired *t*-test showed significant decrease for the Asian participants when evaluated against Omicron/XBB.1-specific GZB+, CD154+, CD69+, and CD4+ T cell expression as compared to the Black participants (*p* < 0.001). Data are represented as Median ± 95 CI for the Black (N = 12–13), Caucasian (N = 17), and Asian (N = 14) participants. Significance is noted as *p* < 0.05 *, *p* < 0.01 **, *p* < 0.1 ***.

**Figure A2:**
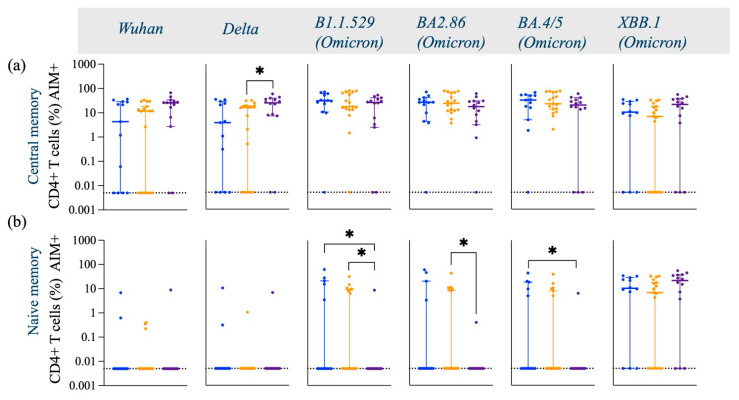

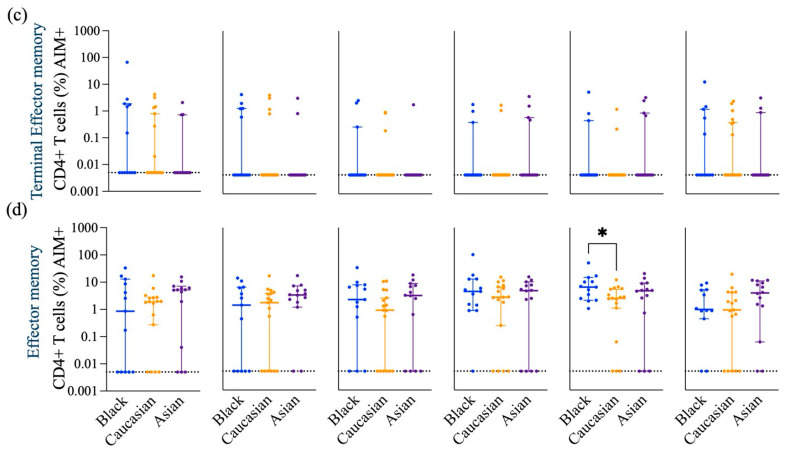
Frequency of Spike-specific AIM+ (CD45RA− CCR7+) CD4+ Central memory, (CD45RA+ CCR7+) Naive memory, (CD45RA+ CCR7−) Terminal effector memory and (CD45RA− CCR7−) Effector memory CD4+ T cells across Wuhan, Delta and Omicron subvariants among Black, Caucasian and Asian participants. (**a**) Mann-Whitney unpaired *t*-test showed no difference among the groups (*p* > 0.05) when evaluated against Wuhan -specific central memory AIM+CD4+ T cell responses. Significant increase was detected in the Asian group as compared to the Caucasians for (**b**) Delta/B1.617.2 -specific central memory AIM+ CD4+ T cell responses. No differences were noted amongst the groups (*p* > 0.05) for Omicron/B1.1.529, Omicron/BA2.86, Omicron/BA4.5 and Omicron/XBB.1—specific CD45RA− CCR7+ CD154+ CD69+ CD4+ T cell responses. (**b**) Mann-Whitney unpaired *t*-test showed no difference among the groups (*p* > 0.05) when evaluated against Wuhan and Delta/B1.617.2 -specific naïve memory AIM+ CD4+ T cell responses. Significant decrease was detected in the Asian group as compared to the Caucasians and the Black participants (*p* < 0.05) for Omicron/B1.1.529—specific naïve memory AIM+ CD4+ T cell responses. Significant decrease was observed in the Asian group for Omicron/BA2.86 as compared to the Caucasians (*p* < 0.05) and for Omicron/BA4.5 specific as compared to the Black participants (*p* < 0.05). No differences noted amongst the groups (*p* > 0.05) when tested against Omicron/XBB.1—specific naïve memory AIM+ CD4+ T cell responses. (**c**) Mann-Whitney unpaired *t*-test showed no difference among the groups (*p* > 0.05) when evaluated against Wuhan, Delta/B1.617.2, Omicron/B1.1.529, Omicron/BA2.86, Omicron/BA4.5 and Omicron/XBB.1—specific terminal effector memory AIM+ CD4+ T cell responses. (**d**) Mann-Whitney unpaired *t*-test showed no difference among the groups (*p* > 0.05) when evaluated against Wuhan, Delta/B1.617.2, Omicron/B1.1.529 and Omicron/BA2.86—specific effector memory AIM+ CD4+ T cell responses. Significant increase was detected in the Black group as compared to the Caucasian group when tested against Omicron/BA4.5—specific effector memory AIM+ CD4+ T cell responses. No significant differences were detected among the groups (*p* > 0.05) for Omicron/XBB.1—specific effector memory AIM+ CD4+ T cell responses. Data is represented as Median ± 95CI for the Black (N = 12–13), Caucasian (N = 17) and Asian (N = 14) participants. Significance is noted as *p* < 0.05 *.

**Figure A3:**
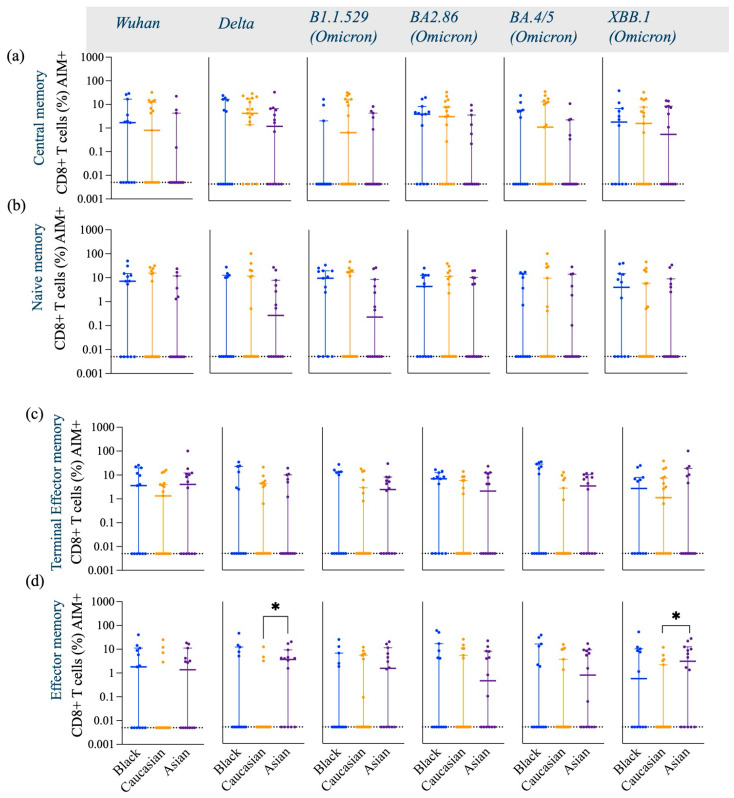
Frequency of Spike-specific AIM+ (CD45RA− CCR7+) CD8+ Central memory, (CD45RA+ CCR7+) Naive memory, (CD45RA+ CCR7−) Terminal effector memory and (CD45RA− CCR7−) Effector memory CD8+ T cells across Wuhan, Delta and Omicron subvariants among Black, Caucasian and Asian participants. (**a**) Mann-Whitney unpaired *t*-test showed no differences among the groups (*p* > 0.05) when evaluated against Wuhan, Delta/B1.617.2, Omicron/B1.1.529, Omicron/BA2.86, Omicron/BA4.5 and Omicron/XBB.1—specific central memory AIM+ CD8+ T cell responses. (**b**) Mann-Whitney unpaired *t*-test showed no differences among the groups (*p* > 0.05) when evaluated against Wuhan, Delta/B1.617.2, Omicron/B1.1.529, Omicron/BA2.86, Omicron/BA4.5 and Omicron/XBB.1—specific naïve memory AIM+ CD8+ T cell responses. (**c**) Mann-Whitney unpaired *t*-test showed no difference among the groups (*p* > 0.05) when evaluated against Wuhan, Delta/B1.617.2, Omicron/B1.1.529, Omicron/BA2.86, Omicron/BA4.5 and Omicron/XBB.1—specific terminal effector AIM+ CD8+ T cell responses. (**d**) Mann-Whitney unpaired *t*-test showed no difference among the groups (*p* > 0.05) when evaluated against Wuhan—specific effector memory AIM+ CD8+ T cell responses. Significant increase was detected in the Asian group as compared to the Caucasian group (*p* < 0.05) when tested against Delta/B1.617.2—specific effector memory AIM+ CD8+ T cell responses. No differences were noted amongst the groups (*p* > 0.05) when tested against Omicron/B1.1.529, Omicron/BA2.86 and Omicron/BA4.5—specific effector memory AIM+ CD8+ T cell responses. Significant increase was detected in the Asian group as compared to the Caucasians (*p* < 0.05) when tested against Omicron/XBB.1—specific effector memory AIM+ CD8+ T cell responses. Data is represented as Median ± 95CI for the Black (N = 12–13), Caucasian (N = 17) and Asian (N = 14) participants. Significance is noted as *p* < 0.05 *.

**Figure A4:**
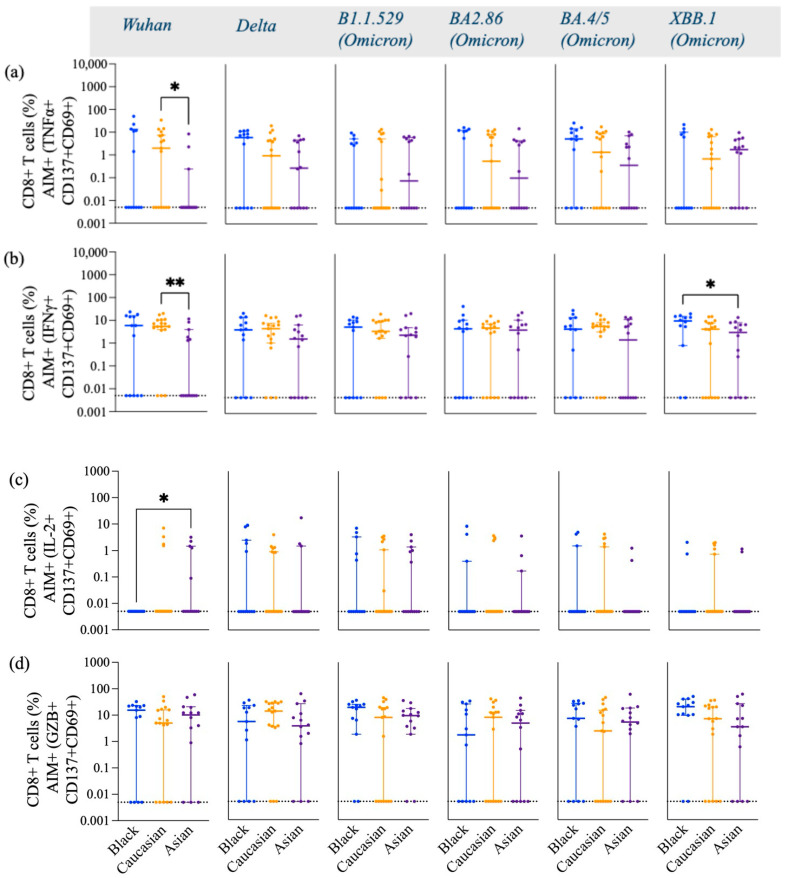
Frequency of Spike-specific AIM+ TNFα+, IFNγ+, IL-2+ and GZB+ CD8+ T cells across Wuhan, Delta and Omicron subvariants among Black, Caucasian and Asian participants. (**a**) Mann-Whitney unpaired *t*-test showed significant difference between the Caucasian and the Asian groups for (**a**) Wuhan-specific (*p* < 0.05) but no difference was detected among the groups when assessed for Delta/B1.617.2, Omicron/B1.1.529 (*p* > 0.05), Omicron/BA2.86, Omicron/BA4.5 and, Omicron/XBB.1—specific TNFα+ CD137+CD69+ CD8+ T cells expression. (**b**) Mann-Whitney unpaired *t*-test showed significant difference between the Caucasian and the Asian groups for Wuhan-specific (*p* < 0.01) but no difference was detected among the groups (*p* > 0.05) when assessed for Delta/B1.617.2, Omicron/B1.1.529, Omicron/BA2.86 and, Omicron/BA4.5. Significant decrease was noted in the Asian group when compared to the Black participants’ group for Omicron/XBB.1—specific IFNγ+ CD137 + CD69+ CD8+ T cell responses. (**c**) Mann-Whitney unpaired *t*-test showed significant difference between the Black and the Asian groups for Wuhan-specific (*p* < 0.05) but no difference was detected among the groups (*p* > 0.05) when assessed for Delta/B1.617.2, Omicron/B1.1.529, Omicron/BA2.86, Omicron/BA4.5 and Omicron/XBB.1—specific IL-2+ CD137+CD69+ CD8+ T cells expression. (**d**) Mann-Whitney unpaired *t*-test showed no significant difference among the groups (*p* < 0.05) for Wuhan, Delta/B1.617.2, Omicron/B1.1.529 (*p* > 0.05), Omicron/BA2.86, Omicron/BA4.5 and Omicron/XBB.1—specific GZB+ CD137+CD69+ CD8+ T cells expression. Data is represented as Median ± 95CI for the Black (N = 12–13), Caucasian (N = 17) and Asian (N = 14) participants. Significance is noted as *p* < 0.05 *, *p* < 0.01 **.

Interestingly, the Asian group showed higher expression levels of central memory AIM+ and CD4+ T cells against the Delta-specific strain as compared to their Caucasian counterparts (*p* < 0.05) [Figure A2a], and the Black participants showed a significantly higher effector memory AIM+ and CD4+ T cell Omicron/BA.4/5-specific responses as compared to the Caucasians (*p* < 0.05) [Figure A2d]. Frequencies of AIM+ and CD8+ T cells were expressed similarly amongst all ethnic groups (*p* > 0.05) across all strains evaluated (Figure 3c). Interestingly, AIM+ and CD8+ T cells exhibited a more diverse repertoire of memory cell subtypes, with terminal effector and central memory populations being more predominant (Figure 3c). Interestingly, the effector memory AIM+ and CD8+ T cell responses were higher for the Asian participants as compared to the Caucasians when evaluated against the Delta (*p* < 0.05) and the XBB.1 (*p* < 0.05) variants [Figure A3d]. For a detailed breakdown of AIM+, CD4+, and CD8+ T memory cell subsets, refer to [Figure A2 and Figure A3]. The functional profile of the spike-specific T cells was evaluated through the frequency of AIM+ cytokine-secreting cells (Figure 4 and Figure A4).

There were no differences in the frequency of TNFα (Figure 4a), IFNγ (Figure 4b), and GZB (Figure 4d) AIM+ secreting-cells against Wuhan and Delta (*p* > 0.05) in any of the three ethnic groups. However, the frequencies of these cytokines were differentially expressed against the Omicron subvariants among the groups. Asian participants displayed a significantly high frequency of Th1 cytokines, including TNFα (Figure 4a), IFNγ+ (Figure 4b), and IL-2+ (Figure 4c) against Omicron subvariants. More specifically, Asian participants had a significantly higher expression of IL-2 against Wuhan (*p* < 0.05) and the Omicron subvariants B.1.1.529 (*p* < 0.05) and BA.4/5 (*p* < 0.05) compared to Caucasian participants (Figure 4c). Asian participants also had a higher number of Omicron-specific (B.1.1.529, *p* < 0.05; BA4/5, *p* < 0.01; XBB.1, *p* < 0.05) TNFα (Figure 4a), and IFNγ+ (B.1.1.529, *p* < 0.05) (Figure 4b)-secreting CD4+ T cells compared to Caucasian participants. Black participants expressed significantly high levels of XBB.1 Spike-specific GZB+- and AIM+-secreting cells compared to the Caucasian participants (*p* < 0.05) and Asian participants (*p* < 0.001) (Figure 4d). Caucasian participants also had a significantly higher frequency of XBB.1-specific GZB+, AIM+, and CD4+ T cells compared to Asian participants (*p* < 0.05) (Figure 4d). No differences were detected among the groups for the GZB-secreting AIM+ and CD8+ T cell subtypes (*p* > 0.05) [Figure A4d], yet Wuhan-specific IFNγ+-secreting CD8+ T cell responses were lower in the Asian participants as compared to the Caucasians (*p* < 0.01) and for the Omicron/XBB.1 specific responses as compared to the Black participants (*p* < 0.05) [Figure A4b]. No differences were detected against the other strains (*p* > 0.05) [Figure A4]. IL-2- and TNFα-secreting AIM+ and CD8+ T cells had overall comparable levels among the groups against most variants. Yet, Asian participants exhibited higher IL-2, AIM+, and CD8+ T cell responses against the Wuhan variant (*p* < 0.05) as compared to the Black participants [Figure A4c] and lower TNFα, AIM+, and CD8+ T cell responses against the same variant (*p* < 0.05) as compared to the Caucasian group [Figure A4a].

### 3.2. Longitudinal Anti-RBD IgG Antibody Titers

**Figure 5:**
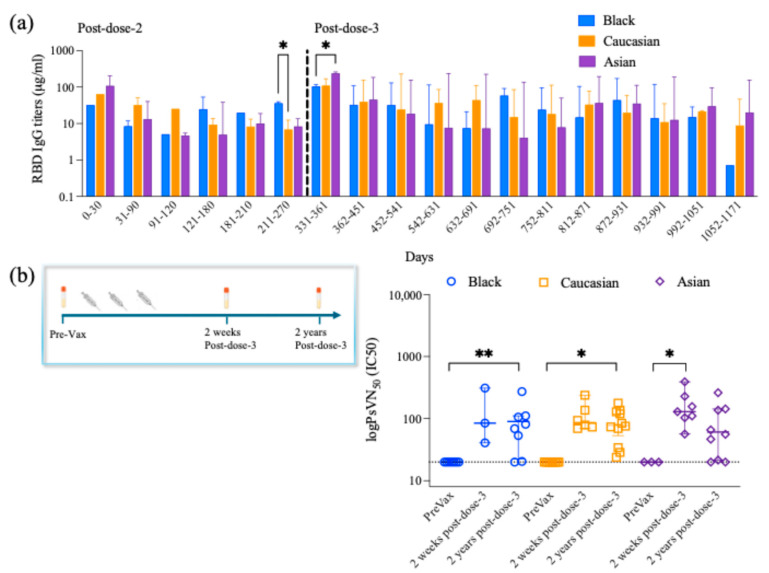
RBD- IgG antibody titer longitudinal responses and neutralizing antibody titers. (**a**) RBD-IgG Antibody titer responses are divided into 30-day increments from post-second and post-third booster doses among the groups for a period between up to 1171 days post-second dose and indicating the time when they received their third booster dose. Two-way ANOVA multiple comparisons with Tukey post-hoc analysis showed no difference for the Ethnicity factor (F_(2,42)_ = 1.182, *p* > 0.05) or Ethnicity × Days (F_(34,125)_ = 0.7114, *p* > 0.05). Tukey post-hoc analysis for the time frames assessed, starting from 0–30 days until 181–210 days post-second dose, showed no differences among the groups, yet black participants showed higher RBD-IgG antibody titer levels as compared to the Caucasian participants (*p* < 0.05) at 211–270 days post-second dose. The Asian participants showed higher levels of RBD-IgG antibody titer as compared to the Black participants at 331–361 days post-second dose after having received their third booster dose. No differences are detected among the groups for the remaining time frames analyzed. Data are represented as Median ± 95 CI for the Black (N = 1–9 per time frame), Caucasian (N = 1–7 per time frame), and Asian (N = 1–7 per time frame) participants. (**b**) Timeline schematic shows that serum Spike-specific neutralizing antibody titers against the ancestral Wuhan were analyzed prior to vaccination (Pre-Vax), at 7–21 days post-dose-3 (two weeks post-dose-3), and 269–800 days post-dose-3 (two years post-dose-3). Kruskal–Wallis with Dunn’s multiple comparisons test showed no differences among the three groups when comparing the 2 weeks with the 2 years post-third-dose time points (*p* > 0.05). Yet, there was a significant decrease in the pre-vaccination and the 2 years post-third-dose time points for the Black participants’ group (*p* < 0.01) and the Caucasian group (*p* < 0.05). Significant decrease was also noted when comparing the pre-vaccination and the 2 weeks post-third-dose time points for the Asian group (*p* < 0.05). Data are represented as Median ± 95 CI for the PreVax (N = 4–10), 2 weeks post-third dose (N = 3–9), and the 2 years post-third dose (N = 13–16) for all ethnic groups. Significance is noted as *p* < 0.05 *, *p* < 0.01 **.

Anti-RBD Spike-specific antibody titers were assessed longitudinally 269–800 days following their last vaccination. High titer was maintained for the first 30 days post-second dose, which was gradually declining until it increased within 331–361 post-second dose, and when the participants had received their third booster dose. Overall, no significant differences (*p* > 0.05) among ethnic groups are detected for most time subdivisions evaluated (Figure 5).

Yet, Black participants showed higher RBD IgG titers as compared to Caucasians (*p* < 0.05) within 211–270 days post-second dose and having received their third booster dose (Figure 5). Also, Asian participants showed higher RBD IgG titers as compared to Black participants (*p* < 0.05) within 331–361 days post-second dose and having received their third booster dose (Figure 5). Spearman’s correlation coefficient analysis showed a positive correlation between the anti-RBD IgG antibody titers and the cTfh when evaluated against the Wuhan (*p* < 0.01) for the Black participants, yet no differences were detected for any of the other groups or any of the other strains evaluated for either group (Table 2).

When evaluating the AIM+, CD4+, and cTfh responses among the groups, no differences among the groups were observed (Table 2). Additionally, there was no significant correlative relationship between anti-RBD antibody titers and AIM+ and CD4+ T cells across all ethnic groups 269–800 days post-vaccination for any strains evaluated (*p* > 0.05) (Table 2). Significance is noted as *p* < 0.05 *, *p* < 0.01 **, *p* < 0.1 ***.

### 3.3. Long-Lived Protective Neutralizing Antibody Titers

Neutralizing antibody titers were assessed 7–21 days following the third vaccination and compared to pre-vaccination levels in each participant (Figure 5). Participants had no detectable neutralizing antibodies against the SARS-CoV-2 Wuhan strain prior to vaccination. However, neutralizing antibodies were detected 269–800 days following the third vaccination (*p* > 0.05) (Figure 5). Specifically, significantly higher neutralizing antibody titers were detected 269–800 days post-dose-3 as compared to pre-vaccination for the Black (*p* < 0.01) and Caucasian participants (*p* < 0.05). Overall, the neutralizing antibody titers are maintained at comparable levels (*p* > 0.05) 7–21 days and 269–800 days post-dose-3 within the ethnic groups (Figure 5). Spearman’s correlation analysis between anti-Wuhan RBD IgG titers and neutralizing antibody titers showed a significant positive correlation in the Black participants’ group (*p* < 0.01), the Caucasian group (*p* < 0.001), and the Asian group (*p* < 0.01). Additionally, the neutralizing antibody titer levels are positively correlated with the cTfh levels in the Black participants against Wuhan (*p* < 0.05), while no significance was detected within the other groups (*p* > 0.05). Spike-specific CD4+ activated T cells and the neutralizing antibody titers showed no significant correlation (*p* > 0.05) against each variant within each group (Table 2).

Table 3 below summarizes the key findings of our study, overall demonstrating well-preserved immunity across all ethnic groups. Arrows pointing upwards used in the table demonstrate high frequency, whereas arrows pointing downwards demonstrate low frequency of the relevant parameter reported.

## 4. Discussion

In this study, we observed robust circulating CD4+- and CD8+-activated T cells across all ethnic groups 2 years after a third COVID-19 vaccination dose. Anti-RBD IgG and neutralizing antibody titers were maintained and were comparable across the ethnic groups, suggesting well-preserved immunity. In line with previous studies that demonstrated that vaccine-induced T cell responses remain largely preserved and vaccine efficacy is less affected in protecting individuals from severe disease and hospitalization [13].

A significant positive correlation between anti-RBD IgG and neutralizing antibody titers across the ethnic groups suggests that these markers may serve as reliable indicators of immune robustness following vaccination. This finding supports growing evidence that anti-RBD antibody levels are strongly associated with neutralizing antibody titers after COVID-19 vaccination or infection. Studies have shown that neutralizing antibodies, which block infection, are closely associated with anti-RBD IgG levels, as both target the RBD [25,26,27]. Since higher neutralizing antibody titers correlate with stronger vaccine-induced protection, our findings reinforce the value of monitoring anti-RBD IgG levels as a potential proxy for neutralizing immunity [28]. While ethnic differences in immune responses have been shown in other studies [6], this study suggests that the correlation between anti-RBD and neutralizing antibodies is consistent across ethnic groups, indicating that antibody-mediated immunity may be similarly predictive irrespective of ethnic background.

When evaluating vaccine-induced cellular responses, Asian participants exhibited a lower frequency of activated Spike-specific CD4+ T cells in response to SARS-CoV-2, particularly against Omicron subvariants. Within the same group, a higher frequency of Th1 cytokine-secreting CD4+ T cells, including TNF-α, IFN-γ, and IL-2, was detected as compared to the Caucasian group. This pattern suggests a potentially more efficient and targeted immune response compared to their Caucasian counterparts, which is in line with other studies having demonstrated increased Th1-targeted responses induced by SARS-CoV-2 vaccines [29]. Th1 and CD4+ T cells play a crucial role in antiviral immunity by promoting cell-mediated responses, enhancing cytotoxic T cell function, and enabling viral clearance [30]. IFN-γ plays a crucial role in preventing viral replication and promoting innate and adaptive immune responses, while TNF-α and IL-2 contribute to T cell proliferation, differentiation, and synchronizing adaptive immune responses [31,32,33].

While Th1 cytokines are vital for an effective antiviral response, excessive release of proinflammatory cytokines, particularly TNF-α and IFN-γ, has been linked to severe COVID-19 symptomatology, including heightened inflammatory response and cytokine storm syndrome [34,35,36,37,38]. This raises the possibility that the heightened proinflammatory T cell profile observed in the Asian-descent participants might contribute to disease severity despite similar antibody protection. It is important to note that breakthrough or prior infections, which enhance both antibody and T cell responses, were present in some participants but could not be fully accounted for. This hybrid immunity could confound interpretations of vaccine-only induced responses. To our knowledge, no prior studies have evaluated long-term circulating SARS-CoV-2 T cell responses alongside neutralizing antibody titers in diverse ethnic populations. Previous research examining immune responses 14–50 days post-second vaccine dose found that South Asian individuals exhibited stronger T cell responses to SARS-CoV-2 spike protein epitopes and higher serum-neutralizing activity than their Caucasian counterparts [6]. However, a separate study tracking immune responses after three mRNA vaccine doses reported that neutralizing antibody levels against Omicron BA.1 and BA.2 declined 3 months post-vaccination, while memory CD4+ T cell responses remained stable [39]. In addition to mRNA-based vaccines, recent studies have explored immune responses to non-mRNA platforms across different populations. Research conducted in Mongolia compared antibody responses among individuals receiving different COVID-19 vaccines, including BNT162b2 (mRNA), ChAdOx1 nCoV-19 (adenoviral vector), Gam-COVID-Vac (adenoviral vector), and BBIBP-CorV (inactivated virus). The study found that BNT162b2 elicited the highest antibody levels, followed by BBIBP-CorV, Gam-COVID-Vac, and ChAdOx1 nCoV-19. Notably, antibody titers were significantly increased in individuals with both vaccination and prior SARS-CoV-2 infection [40]. Similarly, a study involving Egyptian healthcare workers assessed neutralizing antibody titers in response to various vaccines, including mRNA vaccines (BNT162b2 and mRNA-1273), an adenoviral vector vaccine (ChAdOx1), and an inactivated vaccine (Sinovac). The findings indicated that mRNA vaccines elicited higher neutralizing responses overall, though variability existed based on vaccine type [41]. These discrepancies across both ethnic groups and vaccine platforms highlight the potential influence of factors such as genetics, diet, microbiome composition, environmental exposures, and pre-existing health conditions on immune responses. Due to these findings, it is crucial to determine whether these immune variations translate into disparities in COVID-19 severity and long-term vaccine efficacy across ethnic groups. Understanding these differences could provide valuable insights for personalized vaccine strategies and targeted interventions.

Our cohort’s limited sample size and heterogeneity in vaccine types, timing post-vaccination, and infection history restrict generalizability and preclude detailed subgroup analyses, such as within Asian subpopulations. We explicitly acknowledge that grouping diverse Asian subgroups together may mask important biological and environmental differences affecting immune responses. Larger, ethnically stratified studies with careful documentation of infection status are urgently needed.

Despite these promising insights, our study has several limitations. Small sample size, variability in the type of vaccines received, differences in sample collection timing post-third dose, and the inclusion of individuals with prior SARS-CoV-2 infections or breakthrough infections, symptomatic or asymptomatic, may contribute to variability in T cell activation and function. These factors make it challenging to disentangle vaccine-induced immunity from hybrid immunity, especially due to the extended time span of sample collection. We also explicitly acknowledge that grouping diverse Asian subgroups together may mask important biological and environmental differences affecting immune responses. However, our findings suggest potential variations in immune response based on ethnicity at a T-cellular level. Further research is needed to confirm these results and clarify the underlying mechanisms. Due to these complexities, identifying reliable biomarkers of T cell responses is essential, as they may serve as better indicators of long-term vaccine efficacy than antibody testing alone. In conclusion, this study provides preliminary evidence for long-lasting cellular immunity and antibody protection across different ethnic groups, highlighting both shared and unique immune-response patterns.

## 5. Conclusions

Our study highlights the sustained and robust cellular and humoral immune responses observed 2 years after a third COVID-19 vaccine dose across diverse ethnic groups. The strong correlation between anti-RBD IgG and neutralizing antibody titers suggests these markers may serve as reliable indicators of lasting vaccine-induced immunity, regardless of ethnicity. Specifically, distinct T cell response patterns, particularly among Asian participants, underline potential ethnic variations in immune activation and cytokine profiles, which may have implications for disease severity and personalized vaccine strategies. While the observed immune durability is encouraging, the complexity of immune responses and their modulation by various biological and environmental factors that our study could not account for requires further investigation. Continued research into long-term immunity, especially at the cellular level, is essential to refine our understanding of vaccine efficacy and inform equity public health strategies in diverse populations.

## Figures and Tables

**Table 1:** Demographics and clinical characteristics of the study.

Characteristics	Black (N = 13)	Caucasian (N = 17)	Asian (N = 14)
Age (years)			
18–30	4	8	9
31–50	6	8	4
>51	3	1	1
Gender			
Males	4	6	4
Females	9	11	10
Vaccine type			
Pfizer	10	11	10
Moderna	4	6	1
AstraZeneca	1	0	2
BBIBP-CorV (Sinopharm)	0	0	1
Pfizer Bivalent	1	0	0
Pre-Immunity			
Naïve	12	15	13
Pre-Immune	1	2	1
Breakthrough Infections			
Positive PCR or self-reported/RBD titer	5	9	6
Pre-existing health conditions			
Asthma	1	4	0
Hypothyroidism	0	1	1
Wolff-Parkinson-White syndrome	0	1	0
Chronic Kidney disease	0	1	0
Hypertension	3	0	0
Hypercholesteraemia	0	0	2
Hypertriglyceridemia	0	0	1
Prescribed Medications			
Levothyroxine	0	1	0
Albuterol	1	0	0
Paxlovid	1	0	0

**Table 2:** Spearman’s Correlation coefficient analysis of measuremenets of RBD IgG Ab titers, Neutralizing Ab titers, CD4+ Activated T cells and cTfh per variant in Black, Caucasian and Asian participants.

		Wuhan	Delta	B1.1.529	BA2.86	BA4/5	XBB.1
**Black participants**							
RBD IgG Ab titers × cTfh	Spearman’s r	0.732	0.102	0.252	0.382	0.189	0.223
	*p value*	0.0098 **	0.757	0.430	0.221	0.557	0.506
RBD IgG Ab titers × CD4+ Activated T cells	Spearman’s r	−0.427	−0.280	−0.308	−0.392	−0.270	−0.219
	*p value*	0.169	0.379	0.331	0.207	0.394	0.515
Neutralizing Ab titers × RBD IgG Ab titers	Spearman’s r	0.741	-	-	-	-	-
	*p value*	0.0078 **	-	-	-	-	-
Neutralizing Ab titers × cTfh	Spearman’s r	0.644	−0.345	−0.005	0.406	0.341	0.220
	*p value*	0.021 *	0.248	0.993	0.170	0.252	0.487
Neutralizing Ab titers × CD4+ Activated T cells	Spearman’s r	−0.291	−0.137	−0.275	−0.160	−0.198	−0.053
	*p value*	0.334	0.656	0.363	0.600	0.513	0.873
**Caucasian participants**							
RBD IgG Ab titers × cTfh	Spearman’s r	0.312	−0.030	0.167	0.213	0.275	0.289
	*p value*	0.208	0.906	0.507	0.396	0.270	0.245
RBD IgG Ab titers × CD4+ Activated T cells	Spearman’s r	0.174	0.103	0.140	0.108	0.159	0.061
	*p value*	0.503	0.694	0.592	0.678	0.540	0.817
Neutralizing Ab titers × RBD IgG Ab titers	Spearman’s r	0.800	-	-	-	-	-
	*p value*	0.0006 ***	-	-	-	-	-
Neutralizing Ab titers × cTfh	Spearman’s r	0.371	0.093	0.416	0.371	0.384	0.330
	*p value*	0.173	0.742	0.123	0.175	0.158	0.229
Neutralizing Ab titers × CD4+ Activated T cells	Spearman’s r	−0.071	−0.168	−0.111	−0.229	−0.218	−0.168
	*p value*	0.803	0.549	0.695	0.409	0.434	0.549
**Asian participants**							
RBD IgG Ab titers × cTfh	Spearman’s r	0.319	0.247	0.332	0.327	−0.189	−0.024
	*p value*	0.246	0.372	0.246	0.233	0.498	0.934
RBD IgG Ab titers × CD4+ Activated T cells	Spearman’s r	0.304	0.206	0.370	0.202	0.302	0.075
	*p value*	0.271	0.459	0.193	0.468	0.271	0.790
Neutralizing Ab titers × RBD IgG Ab titers	Spearman’s r	0.792	-	-	-	-	-
	*p value*	0.0012 **	-	-	-	-	-
Neutralizing Ab titers × cTfh	Spearman’s r	0.298	0.093	0.363	0.138	−0.018	−0.081
	*p value*	0.297	0.748	0.221	0.634	0.954	0.782
Neutralizing Ab titers × CD4+ Activated T cells	Spearman’s r	0.425	0.351	0.432	0.256	0.256	0.167
	*p value*	0.131	0.217	0.141	0.374	0.373	0.565

**Table 3:** Summary table.

Parameter	Black Group	Caucasian Group	Asian Group
**Activated CD4+ T Cells**	↑ (comparable to other groups)	↑ (comparable to other groups)	↓ as compared to Caucasian (*p* < 0.05) in response to Omicron subvariants
**Activated CD8+ T Cells**	↑ (comparable to other groups)	↑ (comparable to other groups)	↑ (comparable to other groups)
**TNF-α secreting Activated CD4+ T Cells**	↑ (comparable to other groups)	↓ as compared to Asian (*p* < 0.05) in response to Omicron subvariant	↑ as compared to Caucasian (*p* < 0.05, *p* < 0.01) in response to Omicron subvariants
**IFN-γ secreting Activated CD4+ T Cells**	↓ as compared to Asian (*p* < 0.05) in response to Omicron subvariant	↑ (comparable to other groups)	↑ as compared to Black (*p* < 0.05) in response to Omicron subvariant
**IL-2 secreting Activated CD4+ T Cells**	↑ (comparable to other groups)	↓ as compared to Asian (*p* < 0.05) in response to Omicron subvariants	↑ as compared to Caucasian (*p* < 0.05) in response to Omicron subvariants
**Anti-RBD IgG Levels**	↑ (comparable to other groups)	↑ (comparable to other groups)	↑ (comparable to other groups)
**Neutralizing Antibody Titers**	↑ (comparable to other groups)	↑ (comparable to other groups)	↑ (comparable to other groups)

## Data Availability

The original contributions presented in the study are included in the Appendix A of this article. Further inquiries can be directed to the corresponding author.

## Abbreviations

The following abbreviations are used in this manuscript:

RBD	Receptor-binding domain
Th1	T helper type 1
AIM	Activation-induced marker assays
ICS	Intracellular cytokine staining
PBMCs	Peripheral Blood Mononuclear Cells
cTfh	T follicular helper cells
GZB	Granzyme B
IFNγ	Interferon-gamma
TNFα	Tumor necrosis factor alpha
IL-2	Interleukin 2
DMEM	Dulbecco’s Modified Eagle Medium

## Appendix A

**Table A1:** Extensive description of participants’ characteristics.

ID	Race	Age	Gender	BTI	Pre-Immunity	Days Post-3-Dose	Vaccine Dose 1	Vaccine Dose 2	Vaccine Dose 3
CCT-014	Asian	51	F	no	no	663	Pfizer	Pfizer	Pfizer
CCT-008	Asian	34	M	yes	no	559	Pfizer	Pfizer	Moderna
CVI-739	Asian	18	F	no	no	666	Pfizer	Pfizer	Pfizer
CVI-753	Asian	20	M	no	no	599	Pfizer	Pfizer	Pfizer
CVI-633	Asian	25	F	yes	no	597	Pfizer	Pfizer	Pfizer
CVI-1019	Asian	27	F	yes	no	485	Pfizer	Pfizer	Pfizer
CVI-914	Asian	29	M	no	no	408	Astrazeneca	Astrazeneca	Astrazeneca
CVI-778	Asian	30	F	no	no	522	Astrazeneca	Astrazeneca	Pfizer
CVI-224	Asian	34	F	no	yes	616	Moderna	Moderna	Moderna
CVI-684	Asian	23	F	yes	no	549	Pfizer	Pfizer	Pfizer
CVI-628	Asian	24	M	yes	no	800	Pfizer	Pfizer	Pfizer
CVI-504	Asian	27	F	no	no	728	Pfizer	Pfizer	Pfizer
STM-002	Asian	36	F	yes	no	665	Pfizer	Pfizer	Pfizer
CVI-678	Asian	39	F	no	no	777	Sinopharm CNBC bibp	Sinopharm CNBC bibp	Sinopharm CNBC bibp
CVI-1001	Black	45	F	no	no	436	Pfizer	Pfizer	Pfizer
CVI-772	Black	21	F	yes	yes	583	Pfizer	Pfizer	Pfizer
CVI-961	Black	66	M	no	no	446	Pfizer	Pfizer	Pfizer
CTR-375	Black	20	F	no	no	534	Moderna	Moderna	Moderna
CCT-025	Black	42	M	no	no	626	Moderna	Moderna	Moderna
CVI-807	Black	55	M	yes	no	255	Pfizer	Pfizer	Moderna
CVI-666	Black	30	F	no	no	269	Pfizer	Pfizer	Pfizer Bivalent
CTR-365	Black	70	F	no	no	674	Pfizer	Pfizer	Pfizer
CVI-987	Black	28	F	no	no	596	Astrazeneca	Astrazeneca	Pfizer
CVI-1032	Black	33	M	yes	no	347	Pfizer	Pfizer	Pfizer
LNC-003	Black	43	F	yes	no	504	Moderna	Moderna	Moderna
P-201	Black	46	F	no	no	>500	Pfizer	Pfizer	Pfizer
CVI-996	Black	36	F	yes	no	683	Pfizer	Pfizer	Pfizer
CCT-060	Caucasian	33	M	no	no	697	Pfizer	Pfizer	Pfizer
CCT-052	Caucasian	34	F	yes	no	652	Pfizer	Pfizer	Pfizer
CVI-926	Caucasian	19	F	yes	no	625	Pfizer	Pfizer	Pfizer
CVI-540	Caucasian	27	F	yes	no	678	Pfizer	Pfizer	Pfizer
CTR-257	Caucasian	25	M	no	no	642	Pfizer	Pfizer	Pfizer
CVI-145	Caucasian	29	M	yes	no	670	Moderna	Moderna	Pfizer
CCT-078	Caucasian	50	F	yes	no	397	Pfizer	Pfizer	Pfizer
CVI-845	Caucasian	25	F	yes	no	620	Moderna	Moderna	Pfizer
CVI-820	Caucasian	25	F	no	no	494	Pfizer	Pfizer	Pfizer
CTR-257	Caucasian	25	M	no	no	558	Pfizer	Pfizer	Pfizer
CCT-031	Caucasian	48	F	no	no	746	Pfizer	Pfizer	Pfizer
CVI-157	Caucasian	29	F	no	yes	495	Pfizer	Pfizer	Pfizer
CCT-050	Caucasian	76	M	yes	yes	698	Moderna	Moderna	Moderna
CCT-002	Caucasian	25	M	no	no	675	Moderna	Moderna	Moderna
CCT-132	Caucasian	32	M	yes	no	658	Moderna	Moderna	Pfizer
CCT-031	Caucasian	48	F	no	no	700	Pfizer	Pfizer	Pfizer
CCT-085	Caucasian	48	F	yes	no	506	Moderna	Moderna	Moderna
CCF1-020	Caucasian	50	F	no	no	707	Pfizer	Pfizer	Pfizer

**Table A2:** Flow cytometry list of antibodies.

Antigen	Clone	Fluorophore	Company	Panel	Catalogue Nb.	Dilution
Viability dye	-	Zombie NIR	Biolegend	surface	423105	1:1,250
CD8	SK1	SB550	Biolegend	surface	344760	1:125
PD1	EH12.1	BB515	BD Bioscience	surface	564494	1:10
CD45RA	HI100	PerCP	Biolegend	surface	304156	1:25
CD154 (CD40L)	24-31	APC	Biolegend	surface	310810	1:50
CCR7	2-L1-A	APCR700	Biolegend	surface	566766	1:25
CD19	HIB19	APCEF780	Invitrogen	surface	50-161-52	1:25
CD14	61D3	APCEF780	Invitrogen	surface	47-0149-42	1:10
CD3	UCHT1	BV570	Biolegend	surface	300436	1:10
CXCR5	J252D4	PE-DAZZLE	Biolegend	surface	356928	1:50
CD4	SK3	cFluor YG 584	Cytek	surface	R7-20041	1:100
CD134 (OX40)	Ber-ACT35	PECY7	Biolegend	surface	350012	1:250
IL2	MQ1-17H12	PERCP-EF 710	Invitrogen	intracellular	50-161-00	1:50
IFNG	4SB3	BV711	Biolegend	intracellular	502540	1:25
TNF	MAB11	BV785	Biolegend	intracellular	502948	1:25
GZB	QA16A02	PE-CY5	Biolegend	intracellular	372226	1:100
CD137	4B4-1	BV750	Biolegend	intracellular	309844	1:50
CD69	FN50	BV510	Biolegend	intracellular	310936	1:25
